# Supplementary material for: Lignin impairs Cel7A degradation of in vitro lignified cellulose by impeding enzyme movement and not by acting as a sink
Source: Biotechnol Biofuels Bioprod. 2024 Jan 19;17:7. doi: 10.1186/s13068-023-02456-3 (PMC10799419; doi:10.1186/s13068-023-02456-3)
Supplement: Supplementary file 1 — Additional file 1: Figure S1. (Related to Fig. 2): All CA concentrations used in data set for lignin polymerized onto acetobacter cellulose in vitro. First column: cellulose only; second column: lignin only. Third through fifth columns: cellulose with lignin polymerized from different concentrations of CA. Top row are interference reflection micrographs, scale bar = 10 μm. Bottom row are scanning electron micrographs, scale bar = 100 nm. Figure S2. (Related to Fig. 2): Lignin generated in vitro deposits heterogeneously onto acetobacter cellulose. Scanning electron micrograph of 3 mM CA sample, with lignin false-colored yellow. Areas in the middle of the image display highly lignified regions of cellulose with sheets of lignin (shown in yellow) covering significant areas of the cellulose surface In contrast, areas in the upper left and bottom right of the image contain less lignin on the cellulose surface, with some regions appearing to have nearly no visible lignin and appearing similar to the cellulose-only samples. Figure S3. Pure in vitro polymerized lignin has similar Cel7A binding properties to lignin polymerized onto cellulose. Lignin was polymerized in the absence of cellulose, following the same procedures as the lignocellulose samples. A drop of lignin solution and a drop of purified cellulose solution were then placed together onto a glass slide. A flow cell was constructed and placed inverted in an oven at 65 °C for 30 min to dry the lignin and cellulose onto the cover slip, and then BSA was flowed into the chamber to prevent nonspecific binding of Cel7A to the glass surface. Qdot-labeled Cel7A was then injected into the flow cell, binding locations were visualized for 500 s in TIRF, and then Basic fuchsin was introduced to fluorescently label the lignin. An image of Cellulose and lignin visualized via IRM is shown on the left (panel A), and an image of Qdot-labeled Cel7A and fluorescently labeled lignin visualized by TIRF is shown in the right (panel B). [file 13068_2023_2456_MOESM1_ESM.pdf]

Lignin impairs Cel7A degradation of in vitro lignified cellulose by impeding enzyme movement and not by acting as a sink

Zachary K. Haviland, Daguan Nong, Nerya Zexer, Ming Tien, Charles T. Anderson, William O. Hancock

Supplemental Figures

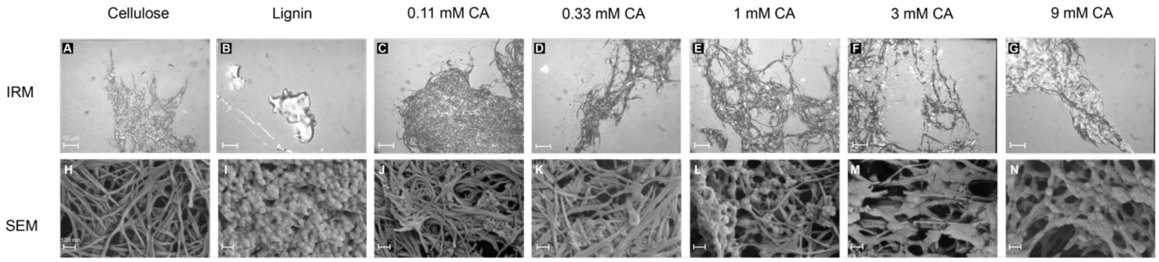

Figure S1 (Related to Figure 2): All CA concentrations used in data set for lignin polymerized onto acetobacter cellulose *in vitro*. First column: cellulose only; second column: lignin only. Third through fifth columns: cellulose with lignin polymerized from different concentrations of CA. Top row are interference reflection micrographs, scale bar = 10 μm. Bottom row are scanning electron micrographs, scale bar = 100 nm.

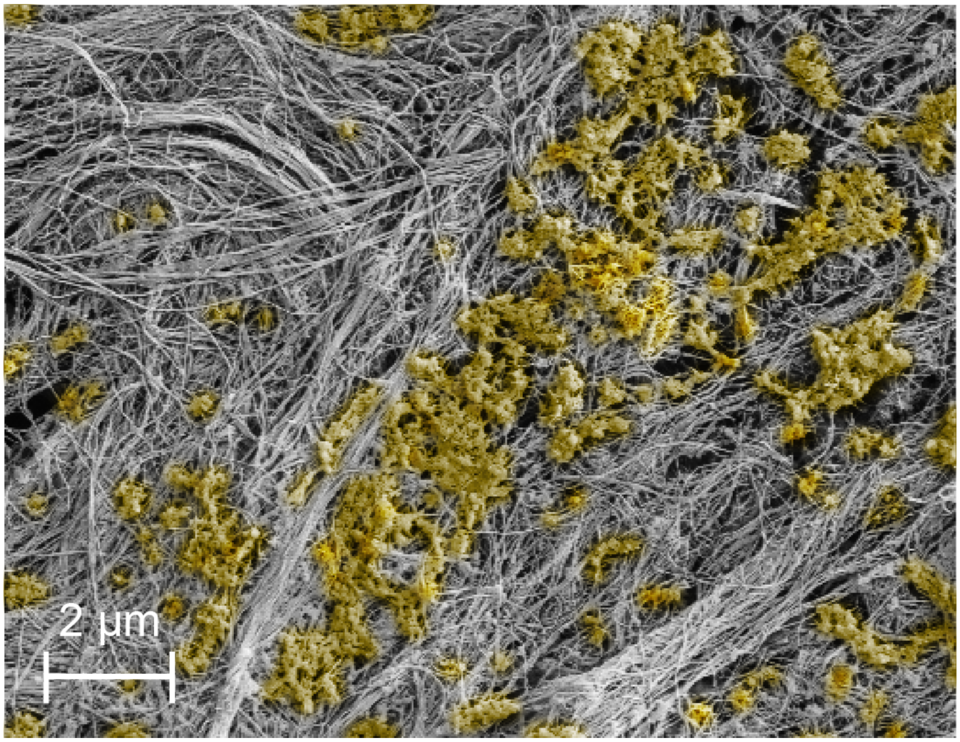

Figure S2 (Related to Figure 2): Lignin generated *in vitro* deposits heterogeneously onto acetobacter cellulose. Scanning electron micrograph of 3 mM CA sample, with lignin false-colored yellow. Areas in the middle of the image display highly lignified regions of cellulose with sheets of lignin (shown in yellow) covering significant areas of the cellulose surface In contrast, areas in the upper left and bottom right of the image contain less lignin on the cellulose surface, with some regions appearing to have nearly no visible lignin and appearing similar to the cellulose-only samples.

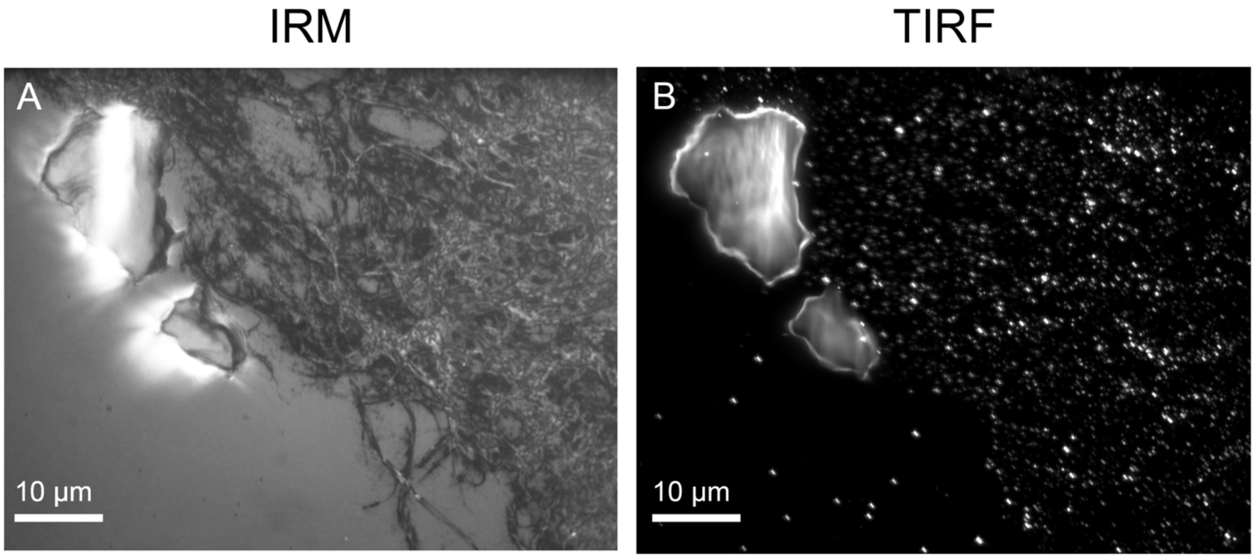

Figure S3: Pure in vitro polymerized lignin has similar Cel7A binding properties to lignin polymerized onto cellulose. Lignin was polymerized in the absence of cellulose, following the same procedures as the lignocellulose samples. A drop of lignin solution and a drop of purified cellulose solution were then placed together onto a glass slide. A flow cell was constructed and placed inverted in an oven at 65°C for 30 minutes to dry the lignin and cellulose onto the cover slip, and then BSA was flowed into the chamber to prevent nonspecific binding of Cel7A to the glass surface. Qdot-labeled Cel7A was then injected into the flow cell, binding locations were visualized for 500 seconds in TIRF, and then Basic fuchsin was introduced to fluorescently label the lignin. An image of Cellulose and lignin visualized via IRM is shown on the left (panel A), and an image of Qdot-labeled Cel7A and fluorescently-labeled lignin visualized by TIRF is shown in the right (panel B). Note that Cel7A preferentially binds to the cellulose with minimal binding to the lignin.

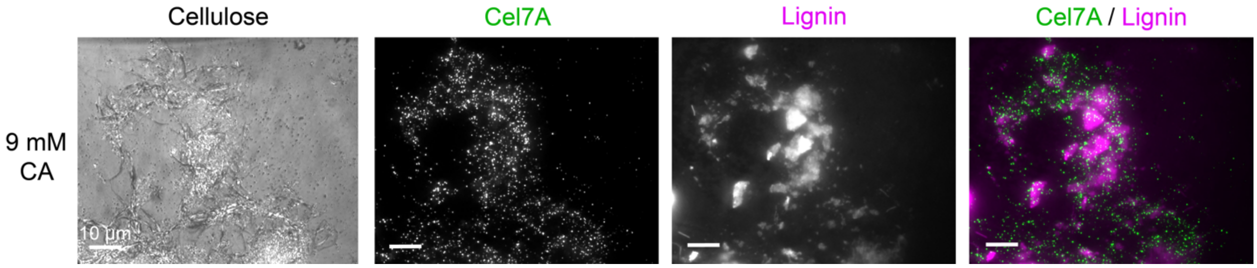

Figure S4 (Related to Figure 3): BSA reduces the binding of Cel7A to lignin. 9 mM CA lignocellulose was adsorbed to the slide without the addition of BSA to the flow cell to determine if BSA affects Cel7A binding to lignin. Binding locations of Qdot-labeled Cel7A were recorded for 50 seconds before being washed out and Basic Fuchsin was added to determine locations of lignin deposition. A Pearson's correlation coefficient of 0.037 was calculated, as described in Methods. This value indicates no correlation between Cel7A binding and lignin, which differs from previous results with BSA added to the flow cell as shown in Figure 3. This shows the BSA washes may affect the Cel7A binding to lignin, but the lignin still does not appear to act as a sink where a larger positive correlation would be expected.

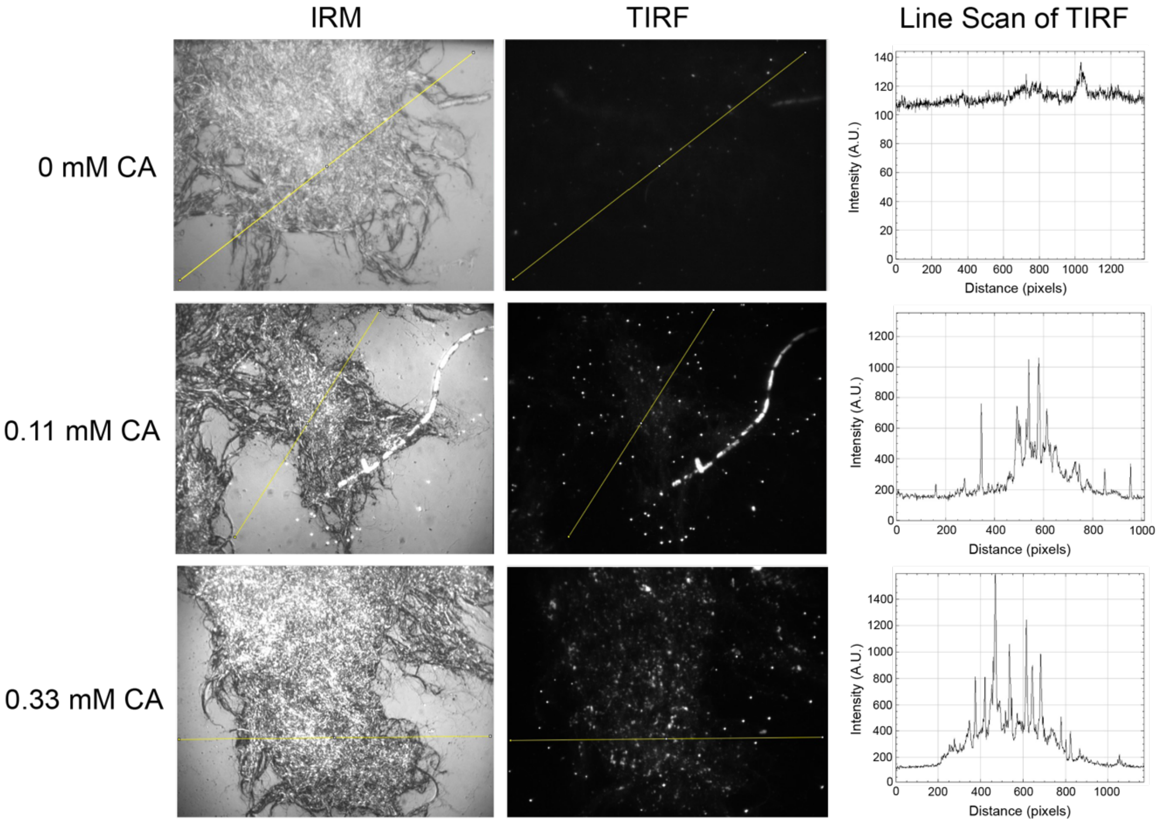

Figure S5 (Related to Figure 5): Line scans across the lignocellulose surfaces show an increase in Basic Fuchsin fluorescent signal in the TIRF channels on the lignified cellulose samples compared to cellulose-only samples. Yellow lines in the IRM and TIRF images show the location of the line scan for each sample. The fluorescence intensity across the cellulose surface for the cellulose-only sample is similar to the intensity on the glass surface, indicating no lignin is present in the sample. The 0.11 mM and 0.33 mM CA samples display an increase in fluorescence intensity across the lignocellulose surface compared to the glass surface, signifying the presence of a thin film of lignin on the cellulose surface. The line scans for the lignified samples also show periodic spikes, corresponding to lignin aggregates that can be seen by eye in the fluorescence image.
